# Editorial—Immune reset in sight: CAR-T cell therapy in autoimmune disease

**DOI:** 10.1016/j.ero.2025.06.003

**Published:** 2025-07-09

**Authors:** Melanie Hagen, Georg Schett, Andreas Wirsching

**Affiliations:** 1 Department of Internal Medicine 3 – Rheumatology and Immunology, Friedrich-Alexander-Universität (FAU) Erlangen-Nürnberg and Universitätsklinikum Erlangen, Erlangen, Germany; 2 Deutsches Zentrum Immuntherapie (DZI), FAU Erlangen-Nürnberg and Universitätsklinikum Erlangen, Erlangen, Germany

The treatment landscape for autoimmune diseases is entering a transformative phase. For decades, our therapeutic strategies have relied predominantly on broad immunosuppression—seeking to attenuate immune hyperactivity while avoiding excessive compromise of host defence. Although this approach has enabled disease control for many patients, it mostly requires permanent immunosuppressive therapy. Recent advances are now beginning to challenge this paradigm, offering the prospect of not just dampening the immune response, but selectively reprogramming it—potentially leading to improvement of disease burden without the need for further immunosuppression or even a functional cure.

Chimeric antigen receptor (CAR) T cell therapy, initially developed for the treatment of B cell malignancies [1,2], is emerging as a promising and innovative tool in the field of autoimmunity. By enabling deep and targeted depletion of pathogenic B cells, CD19-directed CAR-T cells can induce profound and durable immunologic effects [[3], [4], [5]]. This shift—from chronic suppression of the immune system to a single infusion resulting in a presumable immune reset—represents a conceptual breakthrough for diseases previously deemed refractory to conventional therapies.

In this issue of EULAR Rheumatology Open, the study by Pecher et al. contributes important early clinical data to this rapidly evolving field. Five patients with systemic sclerosis (SSc) and severe organ involvement were treated with CD19 CAR-T cell therapy. All patients were not eligible for autologous stem cell transplantation due to comorbidities and severe organ manifestations like interstitial lung disease. Only mild cytokine release syndrome (CRS) and no immune effector cell-associated neurotoxicity syndrome (ICANS) occurred. One patient suffered from a herpes simplex virus infection after 53 days and developed a fatal secondary hemophagocytic lymphohystiocytosis (HLH). It remained unclear, if the infection itself led to the HLH or if the viral infection led to a second stimulation of CAR-expansion with subsequent onset of HLH. Four patients are in a stable status without any signs of disease activity without immunosupressive treatment after a median follow-up of 250 days, even though seroconversion was not achieved in these patients.

Their study is particularly relevant for patients who are ineligible for autologous stem cell transplantation, currently one of the few approaches offering deep disease modification in systemic sclerosis [6,7]. Regarding CRS, ICANS, and hematotoxicity, the data are consistent with current literature. Severe complications after CAR-T cell therapy seem less frequent in autoimmune diseases than in haematological malignancies. Reporting a fatal severe adverse event such as hemophagocytic lymphohistiocytosis however reminds us that such scenarios might also occur among rheumatological patients, underscoring the need for careful patient selection, detailed informed consent, and of course close interdisciplinary monitoring—elements that will be crucial in any future implementation pathway. Despite the small cohort size, their findings offer a valuable proof of principle: CAR-T cell therapy may be feasible, and clinically meaningful even in severe autoimmune diseases such as systemic sclerosis, with softening of skin fibrosis, healing of chronic skin ulcerations by improvement of vasculopathy and stabilising lung function. In contrast to published data with systemic lupus erythematosus patients that also achieve seroconversion, SSc patients can reach a significant clinical improvement even without serological remission [8].

The implications for rheumatology are significant. Cellular therapies such as CAR-T cells represent more than a new treatment modality—they reflect a shift in how we conceptualise disease mechanisms and intervention strategies. By targeting the cellular drivers of autoimmunity, these therapies open up new avenues for long-term disease modulation, potentially offering benefits that extend well beyond those achieved through conventional immunosuppressants or biologics [9].

However, this evolving landscape also brings substantial challenges. The manufacturing of autologous CAR-T cells is technically complex, expensive, and currently confined to highly specialised centres. Long-term data on safety, immune system reconstitution, and clinical response in patients with autoimmune disease remain limited. Moreover, questions around scalability, accessibility, and healthcare system integration must be addressed before these therapies can reach broader clinical practice.

Encouragingly, ongoing innovation is addressing many of these concerns. Alternative CAR targets (eg, B cell maturation antigen [BCMA]), off-the-shelf allogeneic CAR-T platforms, and T cell redirecting bispecific antibodies are being actively investigated [[10], [11], [12], [13], [14], [15], [16], [17]]. While most data exist for autologous CD19-directed CAR-T cell therapy, first data are also available for BCMA-targeted approaches. A case report about BCMA CAR-T cell therapy in a myositis patient relapsing after CD19 CAR-T cell therapy was recently published also showing control of the disease after treatment without any other immunosuppressant. New approaches, especially allogeneic CAR-T or bispecific T cell engagers may simplify logistics, reduce costs, and expand the therapeutic reach of immune cell-based treatments. Importantly, these developments are being accompanied by robust clinical trial activity—an essential prerequisite to ensure that progress is grounded in solid scientific evidence.

As rheumatologists, we find ourselves at a special moment in time: the boundaries between oncology and autoimmunity are becoming increasingly permeable, and tools once limited to cancer therapy are now being reimagined for immune modulation. This convergence brings not only opportunity but also the responsibility (i) to critically assess emerging therapies, (ii) to lead their integration into autoimmune care where appropriate, and (iii) to engage in the necessary infrastructure, regulatory, and ethical frameworks required for safe implementation.

The work by Pecher et al. stands as a timely and forward-looking contribution to this dialogue. It reminds us that the treatment of autoimmune disease may soon move beyond long-standing immune suppression towards long-term improvement of disease burden and even cure without the need for ongoing immunosuppression.

## Competing interests

All authors declare they have no competing interests.
